# Bi-Objective Optimization of Techno-Economic and Environmental Performance of CO_2_ Capture Strategy Involving Two-Stage Membrane-Based Separation with Recycling

**DOI:** 10.3390/membranes15070190

**Published:** 2025-06-24

**Authors:** Nobuo Hara, Satoshi Taniguchi, Takehiro Yamaki, Thuy T.H. Nguyen, Sho Kataoka

**Affiliations:** 1 Integrated Research Center for CCUS Implementation, National Institute of Advanced Industrial Science and Technology (AIST), Central 5, 1-1-1 Higashi, Tsukuba 305-8565, Ibaraki, Japan; 2 Research Institute for Chemical Process Technology, National Institute of Advanced Industrial Science and Technology (AIST), Central 5, 1-1-1 Higashi, Tsukuba 305-8565, Ibaraki, Japan

**Keywords:** CCUS, CO_2_ capture, process design, LCA, multi-objective optimization, membrane-based separation

## Abstract

To effectively implement complex CO_2_ capture, utilization, and storage (CCUS) processes, it is essential to optimize their design by considering various factors. This research bi-objectively optimized a two-stage membrane-based separation process that includes recycling, concentrating on minimizing both costs and CO_2_ emissions. The implemented algorithm combined experimental design, machine learning, genetic algorithms, and Bayesian optimization. Under the constraints of a recovery rate of 0.9 and a produced CO_2_ purity of 0.95, six case studies were conducted on two types of membrane performance: the Robeson upper bound and a tenfold increase in permeability. The maximum value of α*(CO_2_/N_2_), used as a constraint, was adjusted to three levels: 50, 100, and 200. The analysis of the Pareto solutions and the relationship between each design variable and the final evaluation index indicates that electricity consumption significantly impacts operating costs and CO_2_ emissions. The results of the case studies quantitatively clarify that improving the α*(CO_2_/N_2_) results in a greater enhancement of process performance than increasing the membrane’s performance by increasing its permeability. Our bi-objective optimization analysis allowed us to effectively evaluate the membrane’s CO_2_ separation and individual CCUS processes.

## 1. Introduction

To mitigate global warming and create a sustainable society, it is essential to achieve carbon neutrality through carbon capture, utilization, and storage (CCUS) [1]. One approach is the post-combustion capture of CO_2_, where CO_2_ is collected from major emission sources such as power plants, cement plants, and steelworks. Researchers have focused on various methods for capturing CO_2_, including absorption, adsorption, membrane separation, and cryogenic techniques [2]. Among all methods, membrane separation has gained increasing attention due to its ability to operate continuously, eliminate the need for thermal energy, offer scalability, and function without chemical agents [3,4,5,6,7].

Improving the design and optimization of the equipment setups and operating conditions used in CCUS processes, such as CO_2_ recovery via membrane-based separation, is crucial for enhancing their practical implementation [8,9,10]. Improving the design and optimizing equipment setups and operating conditions used in CCUS processes, such as CO_2_ recovery via membrane-based separation, is crucial for promoting practical implementation. Energy consumption, cost, and net present value (NPV) are often utilized as evaluation indicators. However, CO_2_ emissions are also a key evaluation index since CCUS aims for carbon neutrality. Therefore, optimizing multiple factors together is essential. We previously reported the bi-objective optimization of cost and CO_2_ emissions for CO_2_ capture by absorption, as well as a combined process of CO_2_ capture by absorption and methanation [11,12]. We subsequently reported the bi-objective optimization of cost and CO_2_ emissions for a basic single-stage membrane CO_2_ separation process for CO_2_ capture [13]. Our analysis of Pareto solutions revealed a trade-off in the optimization process: increasing the membrane area reduces CO_2_ emissions, but at the expense of increased cost. Conversely, increasing power consumption lowers costs but results in higher CO_2_ emissions. Incorporating bi-objective optimization for cost and CO_2_ emissions into the more feasible two-stage membrane process, thereby defining the ideal equipment setup and operating conditions, is expected to accelerate the deployment of membrane-based CO_2_ separation processes.

A review article highlighted that many studies have concentrated on designing and optimizing membrane-based CO_2_ separation processes [7]. A basic membrane module contains one stage; however, for CO_2_ concentrations of 95% or higher and recovery rates exceeding 90%, multiple stages are usually necessary to meet product specifications [14]. Previous reports on the optimization of membrane processes have been published, and our earlier report summarized the details of these processes [13]. Process optimization can be achieved by adjusting the process’s flow parameters, enhancing the performance of the membrane module, and refining operating conditions through sensitivity analyses that identify key parameters related to the process. Earlier studies of sensitivity analyses revealed a trade-off between membrane area and energy use [14,15,16,17,18]. As alternative approaches evolve, systematic methods for optimizing designs by concurrently adjusting multiple design variables have been reported. Two similar methods optimized the operating conditions using brute force or iterative calculations to lower costs while ensuring consistent process flow and membrane performance [19,20]. Another reported approach involves adjusting the membrane’s performance until it is in line with the Robeson upper bound. Through iterative calculations, the membrane performance, membrane area, and the process operating conditions can be optimized [21,22]. Elsewhere, a superstructure has been developed to enhance the configurations of the process flow, considering the flow paths and the allocation of flow rates [23,24,25,26]. While numerous design and optimization methods have been reported, a systematic approach is required to attain a more practical optimal solution. This approach should include clearly defined boundaries, details of the process’s components, fixed final product specifications, and optimized process design variables.

Several previous reports have analyzed and documented the optimal ranges of membrane permeance and selectivity for designing and optimizing membrane processes. In an optimization of a single-stage membrane process, Van Der Sluijs et al. reported that a CO_2_/N_2_ selectivity of approximately 210 or higher and a permeability of 100 Barrer or higher were required to achieve a CO_2_ purity and recovery rate of at least 0.9 each at set cost target of 32 USD/t-CO_2_ [20]. According to the sensitivity analysis results, Merkel et al. proposed that a CO_2_/N_2_ selectivity of approximately 20 to 150 and a permeance of around 1000 GPU or more would represent the optimal performance range of the membrane [14]. They noted that a CO_2_/N_2_ selectivity of 100 or higher is unsuitable due to the trade-off between low permeability and too large membrane area. Xu et al. indicated, through their analysis of a two-stage membrane process guided by the Robeson upper bound, that a membrane exhibiting high CO_2_ permeance (over 1000 GPU) and moderate CO_2_/N_2_ selectivity (ranging from 40 to 60) is fitting for the first stage [18]. On the other hand, the second stage requires a membrane with high CO_2_/N_2_ selectivity (above 100) and moderate CO_2_ permeance (more than 500 GPU). Roussanaly et al. reported on optimizing the costs of a multi-stage membrane-based CO_2_ separation process by varying the membrane’s performance [22]. They concluded that, to compete with technologically advanced absorption processes utilizing monoethanolamine, CO_2_ permeance must exceed 3 m^3^ (STP) m^−2^ h^−1^ bar^−1^ (approximately 1100 GPU), and CO_2_/N_2_ selectivity must be greater than 65. As mentioned earlier, identifying the ideal performance range of the membrane during the design and optimization of a membrane process can provide crucial insights for developing CO_2_ separation membranes.

Several designs and optimizations for membrane processes aimed at separating CO_2_ from other emissions have been reported previously, and optimal areas for improving membrane performance have been proposed. However, the evaluated variables have primarily focused on cost and energy consumption, with our analysis of a single-stage membrane process being the only approach to specifically use CO_2_ emissions as an evaluation index [13]. In order to consider implementing membrane-based separation processes in CCUS, two-stage membrane processes must be optimized for cost and CO_2_ emissions.

Our research centered on a two-stage membrane process for CO_2_ separation, seeking to reduce costs and CO_2_ emissions through a bi-objective approach. The flow of the process contained two stages and recycling. The performance of the membrane in the first and second stages was established, with the equipment configuration and operational conditions identified as key variables. The calculation method for cost and CO_2_ emissions is the same as in our previous report, and the algorithm for bi-objective optimization from that report (MLB-MOGABO) was used with some modifications [13]. This study evaluated the performance of the membrane used in terms of two trade-off relationships: the Robeson upper bound and a tenfold increase in permeability. Additionally, six case studies were analyzed, during which the upper limit of the ideal separation factor α*(CO_2_/N_2_) was adjusted to 50, 100, and 200. For each case study, we thoroughly analyzed the resulting Pareto solutions to see how they relate to membrane performance.

## 2. Materials and Methods

This section outlines the methodology used for implementing the bi-objective optimization. It includes configuring boundary and process simulation settings, evaluating selected evaluation indices, applying the MLB-MOGABO algorithm, and setting up the case studies. All computations were conducted using Python 3.10.11 on a personal computer equipped with an Intel Core i7-1185G7 processor (maximum clock speed: 4.80 GHz).

### 2.1. Boundary and Process Simulation Settings

**Figure 1:**
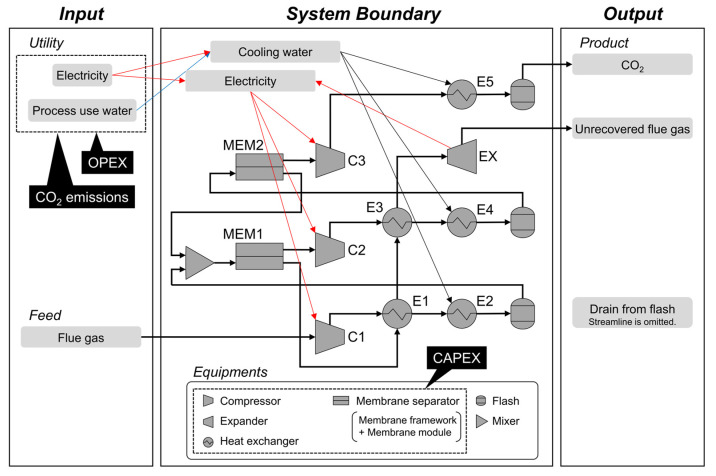
Flow diagram of the process and the system used to evaluate it. The thin red, blue, and black arrows indicate the flow of electricity, process water, and cooling water, respectively.

The investigated process involves a two-stage membrane technique designed to recover CO_2_ from flue gas, as shown in Figure 1. This system takes feed flue gas, electricity, and water as its inputs and produces CO_2_, unrecovered flue gas, and drained water from flashes as its output. The associated equipment codes, types, and settings are detailed in Table 1. The characteristics of the feed gas were defined based on prior research: the flue gas was from a 550 MWe coal-fired power plant and had a flow rate of 79,200 kmol/h at 101 kPa and 57 °C [18]. Its composition was 68.8% N_2_, 13.5% CO_2_, 2.4% O_2_, and 15.3% H_2_O. The design and simulation of the process were carried out using the AVEVA Company’s PRO/II v2022 process simulator [27]. A tolerance of 0.1% was set for calculations during the simulation, with all other settings mirroring those from our earlier study [13].

A built-in module based on the cross-flow method was used to evaluate the membrane separators MEM1 and MEM2 [29]. The permeances of each membrane separator for CO_2_, N_2_, O_2_, and H_2_O were calculated from the ideal separation factor α*(CO_2_/N_2_), which was based on previous studies. The membrane permeances of CO_2_ and N_2_ were adjusted to meet either the Robeson upper bound or a tenfold increase in permeability—as discussed in the settings for the case studies subsection—under the assumption of an effective membrane thickness of 100 nm, which was consistent with prior reports [18,21,30]. Their permeances for O_2_ were then calculated based on the assumption that the ideal separation factor for CO_2_/O_2_ is 0.37 times that of CO_2_/N_2_ [18]. Their permeances for H_2_O were also calculated using an assumed ideal separation factor of 0.8 for CO_2_/H_2_O [18].

### 2.2. Analyses of the Selected Evaluation Indices

The selected evaluation indices were the cost and CO_2_ emissions per unit weight of CO_2_ in the product, and the evaluation parameters used are shown in Table 2. The first estimate of CAPEX was determined by calculating the total module cost (*C_TMi_*) using the equation provided below:

(1) $$\begin{array}{c} C_{TMi}=1.18\times \frac{{CEPCI}_{2021}}{{CEPCI}_{2001}}\times \;C_{p}^{0}{\left(2001\right)}_{i}\times F_{BMi}, \end{array}$$

where *CEPCI* refers to the Chemical Engineering Plant Cost Index; we used the base year values from 2001 and the annual figures for 2021, as outlined in Table 3. *C_p_*^0^ (2001)_i_ indicates the purchase cost of each piece of equipment for the base year 2001, while *F_BMi_* is the bare module factor for each piece of equipment. Both values were calculated according to the parameters specified in Table 1. Following this, the CAPEX per unit weight of CO_2_ in the product was determined using the equation below:

(2) $$\begin{array}{c} CAPEX=\sum \left[\frac{{CRF\times C}_{TMi}}{Product\;flowrate\times Annual\;operation\;hour}\;\right], \end{array}$$

where *i* refers to an item in the comprehensive list of equipment used for the CAPEX evaluation and *CRF* denotes the capital recovery factor, which is used to determine the annual depreciation of the equipment. To estimate the OPEX, we reviewed the utilities costs to determine the cost of cooling water (the electricity and water used in the process). As a result, the expenses for cooling water were listed separately from those utilities. Hence, OPEX was assessed using the equation below:

(3) $$\begin{array}{c} OPEX=\sum \left[Q_{j}\times {Price}_{j}\right], \end{array}$$

where *j* represents an item within the list of utilities; *Q_j_* represents the amount of *j* consumed per unit weight of CO_2_ in the product, which is derived from the process simulation and further evaluations; and *Price_j_* refers to the price of each utility *j*. Therefore, the cost for each unit weight of CO_2_ in the product is evaluated using the following equation:

(4) $$\begin{array}{c} Cost=CAPEX+OPEX. \end{array}$$

The second evaluation index—namely, CO_2_ emissions per unit weight of CO_2_ in the product—was evaluated using the gate-to-gate life cycle assessment (LCA) method. All indirect CO_2_ emissions from the utilities were analyzed under the assumption that the amount of CO_2_ emitted is proportional to the utility’s consumption, as shown in the equation below:

(5) $$\begin{array}{c} {CO}_{2}\;emissions=\sum \left[Q_{k}\times C_{k}\right], \end{array}$$

where *k* indicates an item from the list of utilities (electricity and water); *Q_k_* represents the amount of *k* consumed per unit weight of CO_2_ produced, obtained through process simulations and subsequent evaluations; and *C_k_* denotes the CO_2_ emission factor per unit of each *k*.

### 2.3. Algorithm of MLB-MOGABO

**Figure 2:**
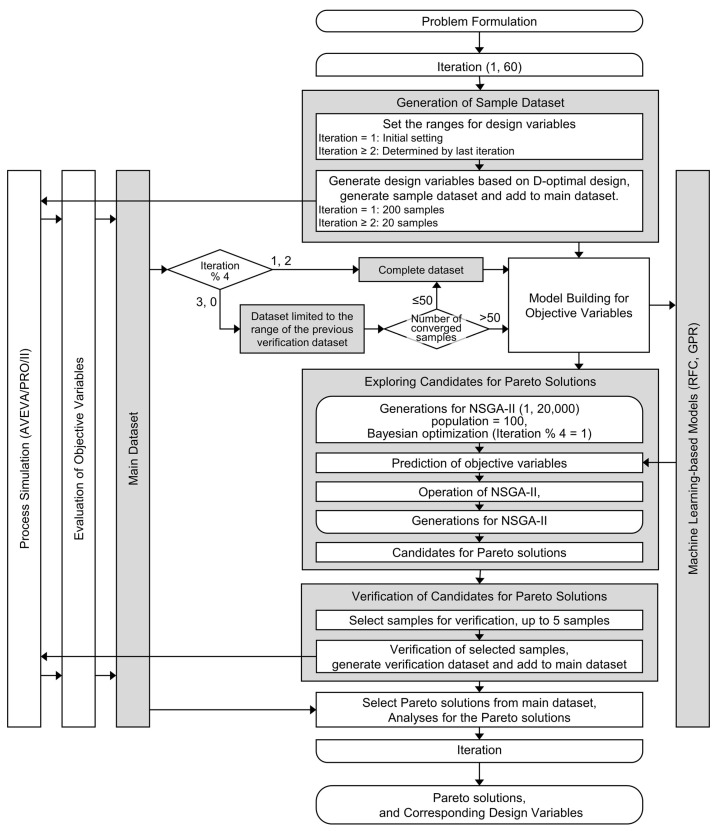
MLB-MOGABO algorithm used in this study.

In the MLB-MOGABO algorithm, the target process underwent evaluation and optimization. A sample dataset was created, a machine learning (ML) model was constructed, and Pareto solution candidates were investigated and confirmed. This process was modified following the adaptive design of experiments (ADoE) approach, which involved adding datasets and updating the models, as illustrated in Figure 2 [35,36].

When initially creating the sample dataset, the ranges for the design variables were determined based on the values listed in Table 3. From iteration 2 onward, these ranges were derived from the previous verification dataset: the minimum and maximum values of the variables were adjusted by multiplying them by 0.8 and 1.2, ensuring they remained within the optimization limits specified in Table 3. Utilizing D-optimal design, sets of design variables were generated within these specified ranges, with 200 generated for iteration 1 and 20 for iteration 2 and subsequent iterations [37]. Process simulations were conducted using the generated design variables, and the objective variables were analyzed afterwards. The main dataset was renewed each iteration by combining all the sample and verification datasets from earlier iterations.

In building the ML model, the complete main dataset was used for every fourth iteration’s first and second instances. The main dataset was limited to match the range of the verification dataset from the previous iteration for the third and fourth instances of every fourth iteration, provided that the count of converged samples exceeded 50, in order to enhance the effectiveness of assessing the Pareto solutions locally. ML models were constructed with ADoE for every iteration, based on the main dataset and the configurations specified in Table 4, employing the scikit-learn library in Python [38]. For Y0, we developed random forest classification (RFC) models by refining hyperparameters using the out-of-bag method [39]. For Y1–Y4, we built Gaussian process regression (GPR) models using the converged data only, optimizing the Gaussian kernel through cross-validation [40].

To explore candidates for the Pareto solution, the elitist nondominated sorting genetic algorithm II (NSGA-II) was employed, alongside the ML models, with the settings outlined in Table 4 [41]. For minimization of the evaluation indices Y3 and Y4, the lower confidence bound Y_Nac_ served as the acquisition function in the Bayesian optimization for the first instance of every fourth iteration [42,43]:

(6) $$\begin{array}{c} Y_{Nac}=Y_{Nave}-{SD}_{N}, \end{array}$$

where *Y_Nave_* and *SD_N_* are the predicted average and standard deviation values generated by the built ML models, respectively. The predicted average value was utilized to minimize the evaluation indices Y3 and Y4 during the second, third, and fourth instances of every fourth iteration. For the explorations, we employed the Platypus-opt library in Python, with the population size set at 100 and a generation capacity of 20,000 [44]. The exploration identified sets of objective variables and their design variables as potential Pareto solution candidates.

A maximum of five candidates were selected based on the maximum hypervolume condition, while verifying the Pareto solution candidates. The hypervolume serves as an evaluation metric for a range of Pareto solutions [45,46], which was analyzed here using the following reference point: (CO_2_ emissions, cost) = (1 t-CO_2_/t-CO_2_, 1 USD/t-CO_2_). Process simulations were then conducted using the design variable dataset made up of the chosen candidates, and the objective variables were obtained in a similar fashion to that used to create the sample dataset. The verification dataset was combined with the main dataset to select Pareto solutions. The iterations mentioned above were implemented up to 60 times.

### 2.4. Settings for the Case Studies

We conduced bi-objective optimizations of six case studies to showcase the potential benefits of enhancing membrane performance; the details of the case studies are presented in Table 5. The membrane’s permeability characteristics were categorized based on two conditions: the Robeson upper bound and a tenfold increase in permeability (P × 10) [30]. The maximum value of α*(CO_2_/N_2_) was adjusted to three levels—50, 100, and 200—resulting in six variations in total. The maximum set value of the ideal separation factor α*(CO_2_/N_2_) was defined as a constraint in the optimization. Bi-objective optimization commenced with the same sample dataset used for cases 1, 2, and 3, where only the Robeson upper bound was considered. In contrast, cases 4, 5, and 6—which involved a tenfold increase in permeability—began with a different sample dataset.

## 3. Results

This section describes the bi-objective optimization of a two-stage membrane-based CO_2_ separation process, and discusses the cost and CO_2_ emissions as evaluation criteria and the obtained Pareto solutions in relation to membrane performance.

### 3.1. Progress in Bi-Objective Optimization

For the optimization using the MLB-MOGABO algorithm, we followed the process outlined in our previous report, which included generating datasets, building models, identifying Pareto solution candidates, validating and selecting Pareto solutions, and performing iterations [13]. The initial sample dataset for cases 1, 2, and 3 included 49 converged solutions out of 200 data points. The initial sample dataset for cases 4, 5, and 6 included 78 converged solutions out of 200 data points.

**Figure A1:**
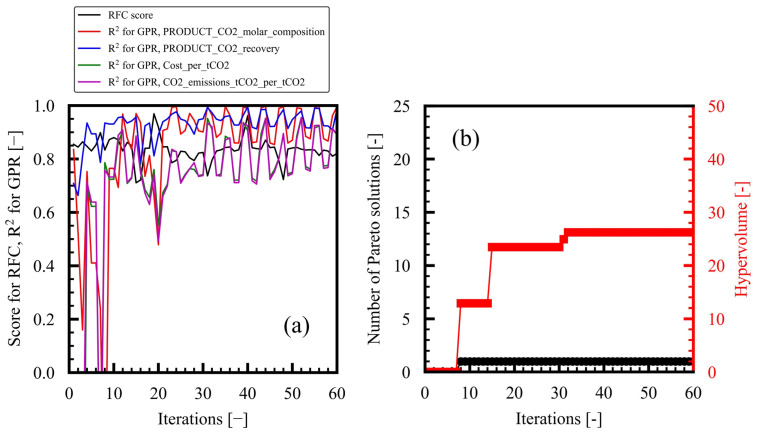
Progress of bi-objective optimization using MLB-MOGABO for case study 1: (**a**) enhancement of the ML models and (**b**) variations in the number of Pareto solutions and hypervolume.

**Figure A2:**
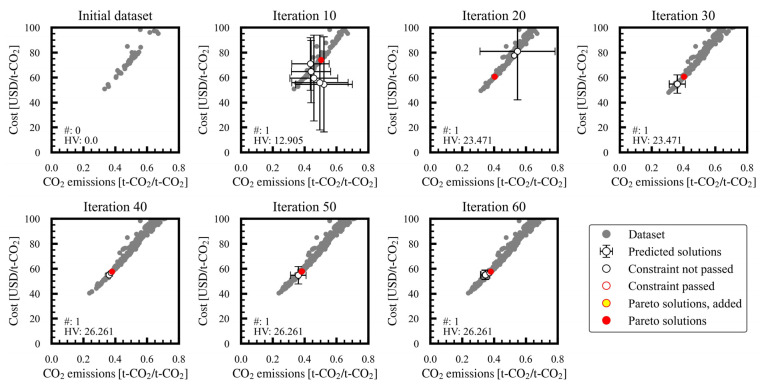
Regular monitoring of the Pareto solutions for case study 1 across the evaluation index space. The open black, red, and yellow-filled red circles are plotted in each iteration when a solution that does not meet the constraints, satisfies the constraints, and new Pareto solutions are found during verification, respectively. The number of Pareto solutions (#) and the hypervolume (HV) are shown together in each figure.

**Figure A3:**
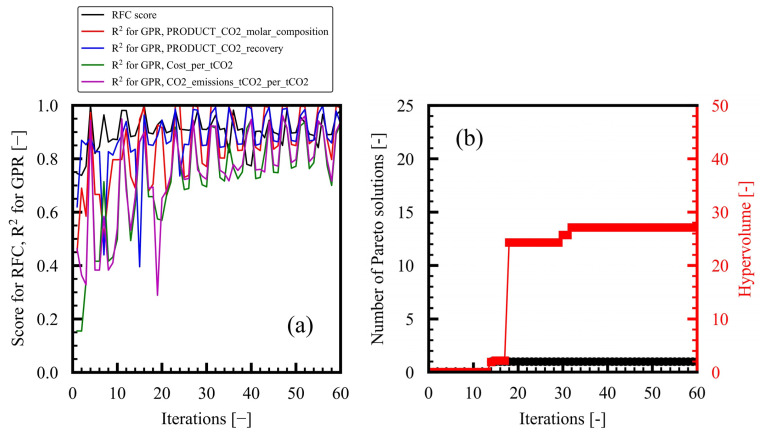
Progress of bi-objective optimization using MLB-MOGABO for case study 2: (**a**) enhancement of the ML models and (**b**) variations in the number of Pareto solutions and hypervolume.

**Figure A4:**
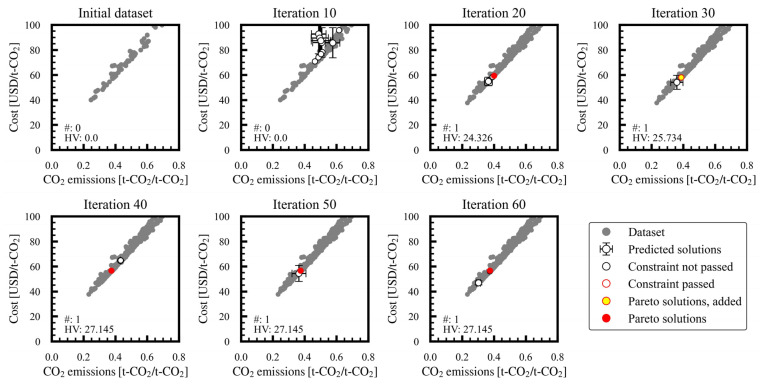
Regular monitoring of the Pareto solutions for case study 2 across the evaluation index space. The open black, red, and yellow-filled red circles are plotted in each iteration when a solution that does not meet the constraints, satisfies the constraints, and new Pareto solutions are found during verification, respectively. The number of Pareto solutions (#) and the hypervolume (HV) are shown together in each figure.

**Figure A5:**
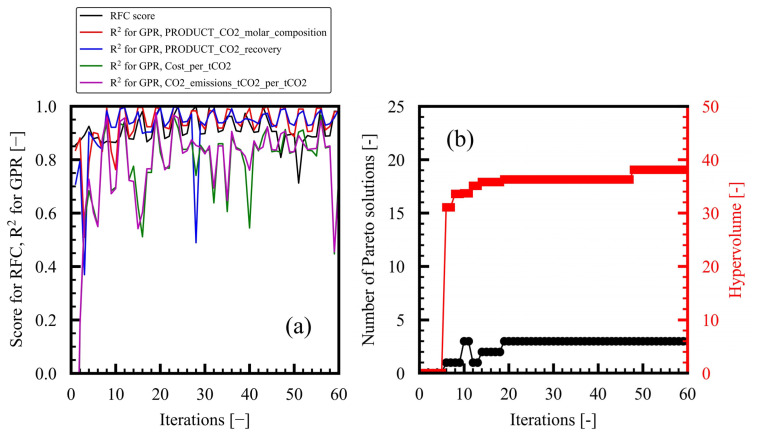
Progress of bi-objective optimization using MLB-MOGABO for case study 3: (**a**) enhancement of the ML models and (**b**) variations in the number of Pareto solutions and hypervolume.

**Figure A6:**
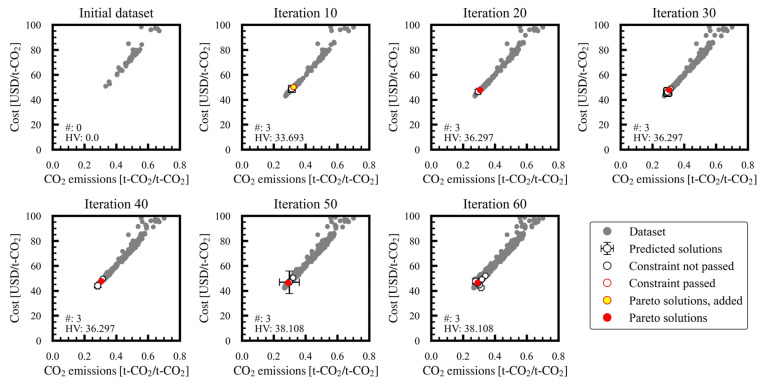
Regular monitoring of the Pareto solutions for case study 3 across the evaluation index space. The open black, red, and yellow-filled red circles are plotted in each iteration when a solution that does not meet the constraints, satisfies the constraints, and new Pareto solutions are found during verification, respectively. The number of Pareto solutions (#) and the hypervolume (HV) are shown together in each figure.

**Figure A7:**
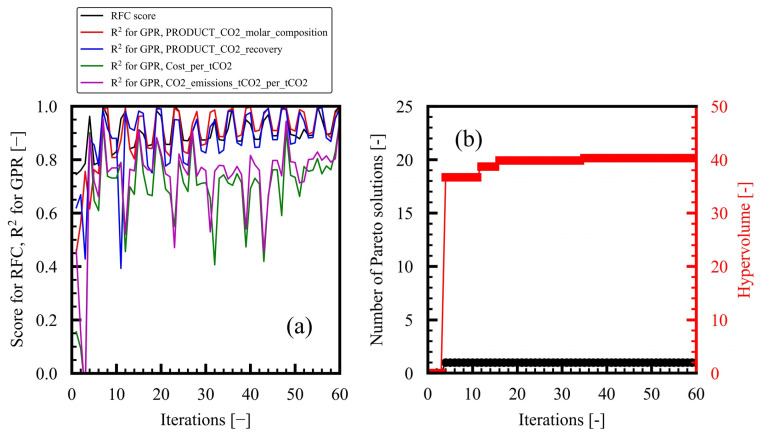
Progress of bi-objective optimization using MLB-MOGABO for case study 4: (**a**) enhancement of the ML models and (**b**) variations in the number of Pareto solutions and hypervolume.

**Figure A8:**
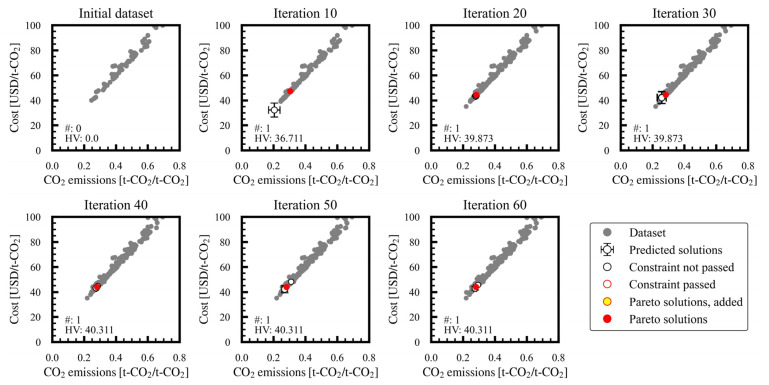
Regular monitoring of the Pareto solutions for case study 4 across the evaluation index space. The open black, red, and yellow-filled red circles are plotted in each iteration when a solution that does not meet the constraints, satisfies the constraints, and new Pareto solutions are found during verification, respectively. The number of Pareto solutions (#) and the hypervolume (HV) are shown together in each figure.

**Figure A9:**
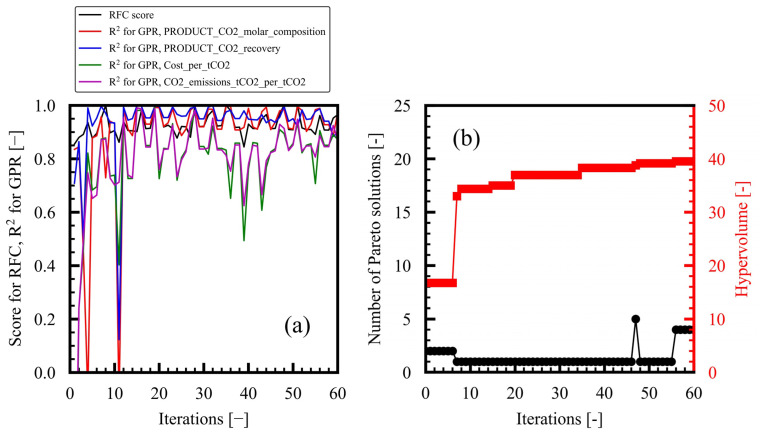
Progress of bi-objective optimization using MLB-MOGABO for case study 5: (**a**) enhancement of the ML models and (**b**) variations in the number of Pareto solutions and hypervolume.

**Figure A10:**
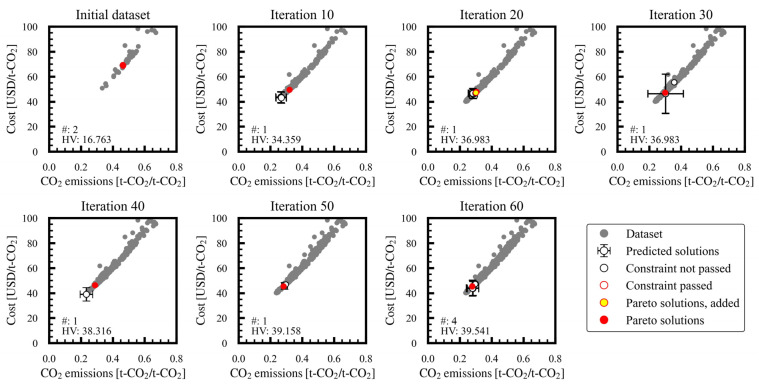
Regular monitoring of the Pareto solutions for case study 5 across the evaluation index space. The open black, red, and yellow-filled red circles are plotted in each iteration when a solution that does not meet the constraints, satisfies the constraints, and new Pareto solutions are found during verification, respectively. The number of Pareto solutions (#) and the hypervolume (HV) are shown together in each figure.

**Figure A11:**
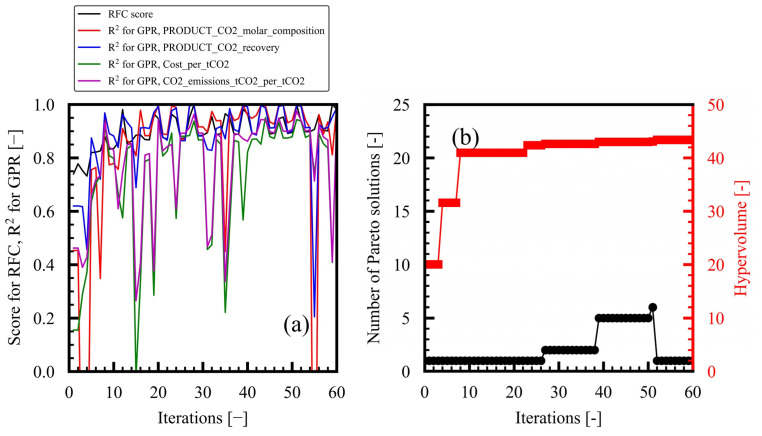
Progress of bi-objective optimization using MLB-MOGABO for case study 6: (**a**) enhancement of the ML models and (**b**) variations in the number of Pareto solutions and hypervolume.

**Figure A12:**
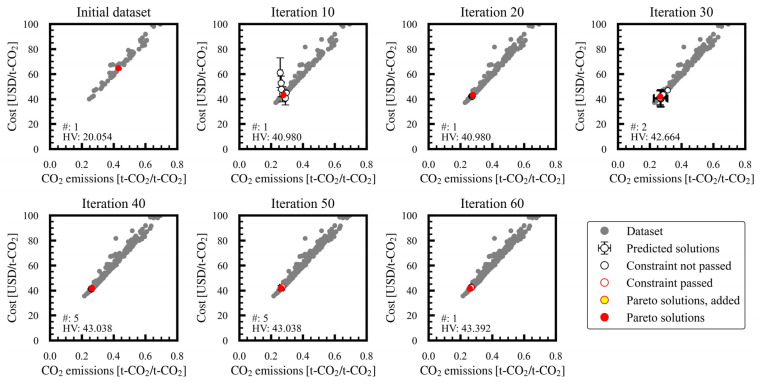
Regular monitoring of the Pareto solutions for case study 6 across the evaluation index space. The open black, red, and yellow-filled red circles are plotted in each iteration when a solution that does not meet the constraints, satisfies the constraints, and new Pareto solutions are found during verification, respectively. The number of Pareto solutions (#) and the hypervolume (HV) are shown together in each figure.

In case 1, data were added as the iterations progressed, and in the final 60 iterations, 669 converged solutions were included out of a total of 1590 data points. The scores generated by the RFC model for each iteration and the R^2^ value of the GPR model were relatively low during the first and second instances of every fourth iteration due to the wide range of design variables included. In contrast, the scores were higher in the third and fourth instances of every fourth iteration because of the narrower range of design variables used, which was limited to match the range of the verification dataset from the previous iteration. Figure A1a illustrates that the overall RFC scores and GPR R^2^ values improved until the final iteration for case 1, ultimately surpassing 0.8. Figure A1b shows that improved Pareto solutions were explored as the iterations progressed, resulting in higher hypervolume values. Iteration 8 was our first to find a Pareto solution, and the number of Pareto solutions did not increase as we went through more iterations. In Figure A2, the regular monitoring demonstrates the effect of bi-objective optimization on case 1. As the iterations continued, the dataset grew and new Pareto solutions were explored, and existing ones were refined. The other case studies underwent the same process, as shown in Figure A3, Figure A4, Figure A5, Figure A6, Figure A7, Figure A8, Figure A9, Figure A10, Figure A11 and Figure A12. Table 5 displays the number of Pareto solutions identified for each case study, which varied from one to four. The values were closely grouped in instances with multiple solutions; therefore, the average of these solutions is used for the following discussion.

### 3.2. Trends in the Pareto Solutions

**Figure 3:**
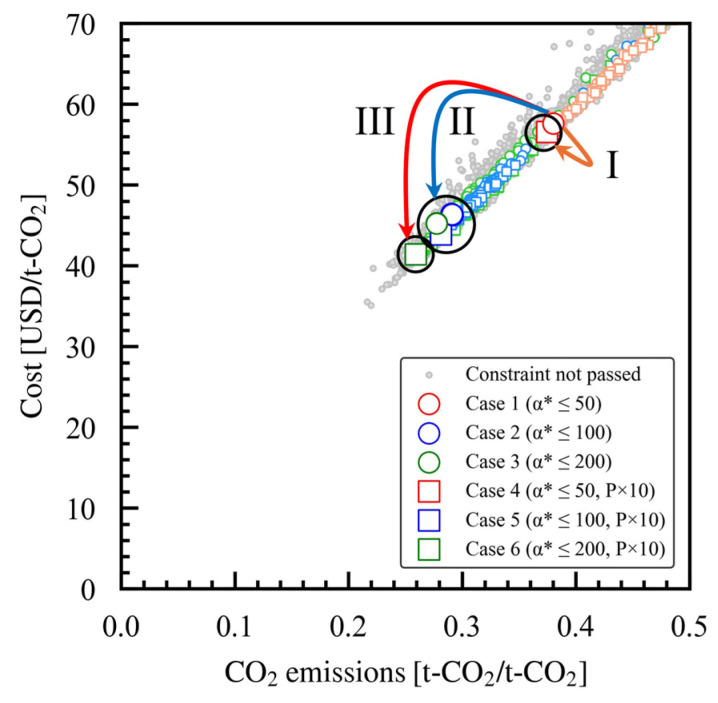
Pareto solutions for case studies 1 through 6, which feature various different membrane performance constraints. In each case study, the larger symbols represent the Pareto solutions, while the smaller symbols indicate the solutions that meet the constraints.

Each case study showed that Pareto solutions had a linear relationship between cost and CO_2_ emissions, as seen in Figure 3. With the maximum setting for α*(CO_2_/N_2_) of 50 (case 1), the cost was approximately 58 USD/t-CO_2_ and the CO_2_ emissions were about 0.38 t-CO_2_/t-CO_2_. Meanwhile, the cost was approximately 57 USD/t-CO_2_, and the CO_2_ emissions were 0.37 t-CO_2_/t-CO_2_ when P × 10 was considered as well (case 4), indicating only a minimal improvement. At the maximum setting value of 100 for α*(CO_2_/N_2_) (case 2), the cost was approximately 46 USD/t-CO_2_ and the CO_2_ emissions were 0.29 t-CO_2_/t-CO_2_. In contrast, with P × 10 considered (case 5) there were slight improvements; the cost was about 44 USD/t-CO_2_ and the CO_2_ emissions were approximately 0.28 t-CO_2_/t-CO_2_. With a maximum α*(CO_2_/N_2_) setting of 200 (case 3), the cost was around 45 USD/t-CO_2_, and the CO_2_ emissions were approximately 0.28 t-CO_2_/t-CO_2_, which is equivalent to that when the maximum α*(CO_2_/N_2_) setting was 100. With the P × 10 considered (case 6), the cost improved to 41 USD/t-CO_2_ and the CO_2_ emissions decreased to about 0.26 t-CO_2_/t-CO_2_. Overall, the Pareto solutions across all case studies reveal that higher α*(CO_2_/N_2_) settings are associated with reduced costs and CO_2_ emissions. At the same maximum α*(CO_2_/N_2_) setting, the choice with a higher permeability (P × 10) resulted in lower costs and CO_2_ emissions.

**Figure 4:**
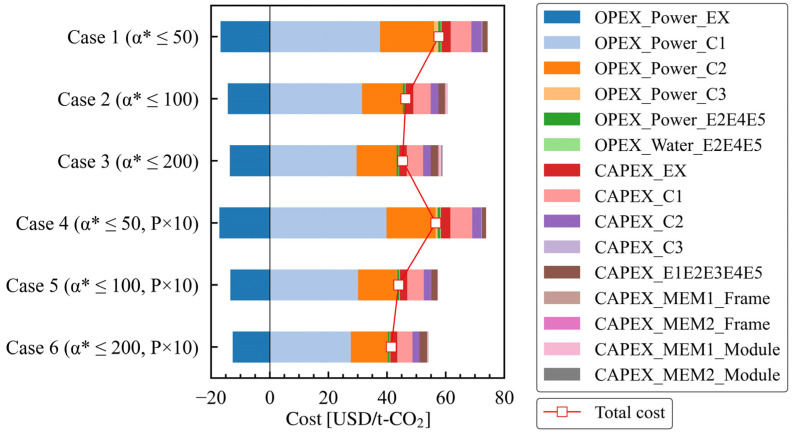
Details of the costs of the Pareto solutions for case studies 1 to 6, which have different membrane performance constraints.

Regarding the breakdown of the Pareto solutions, Figure 4 contains a stacked graph of the cost details. Compressors C1 and C2 supply the feed flow to MEM1 and MEM2, respectively, and due to their high throughput, their electricity costs are substantial. In all case studies, the costs associated with the electricity used by C1 represented more than half of the total costs, while those associated with the electricity used by C2 were about half of that of C1. The electricity costs for C3 were negligible compared to those for C1 and C2. Power recovery was carried out using an expander, Ex, on the retentate side. In all case studies, approximately one-third of the total electricity consumed by C1 and C2 was recovered. Regarding the heat exchangers, their OPEX for electricity and water is minimal. CAPEX accounts for about a quarter of the total cost, with C1, C2, and Ex representing more than half of that amount. The capital expenditure (CAPEX) for the heat exchanger is similar to that of C2 and accounts for a minor portion of the total cost. Conversely, the costs associated with the membrane frame and membrane module represent only a small part of the overall cost. In case study 3, the cost for the membrane module is slightly higher; however, it is less than the CAPEX of C2 and represents a negligible fraction of the overall cost. However, because the vast majority of CO_2_ emissions come from electricity consumption, the emission distribution closely reflects the operational expenditure (OPEX) breakdown, with components C1 and C2 emerging as the primary contributors.

The overall trend in the optimal solutions discovered in this study was clarified earlier in this paper. It was revealed that the use of electricity dominates both evaluation indices: cost and CO_2_ emissions. There is a positive correlation between cost and CO_2_ emissions, as both increase with higher electricity demands. As a result, we did not find a trade-off relationship that would allow for a wide range of Pareto solutions. Instead, this study pinpointed a single optimal solution, as shown in Figure 3. This is in comparison to optimizing a single-stage membrane process using two objectives, as in our previous report, where the optimal solution was a recovery rate of 0.9 and produced CO_2_ purity of 0.9 [13]. In our previous report on the single-stage configuration, optimal costs ranged from 75 to 88 USD/t-CO_2_, with associated emissions of 0.40 and 0.37 t-CO_2_/t-CO_2_, respectively. In this study, the allowable fraction of CO_2_ in the product was set at 0.95, which is higher than in our previous report. However, the optimal cost and CO_2_ emission values achieved were lower than those previously reported, indicating that the two-stage membrane process outperforms the single-stage configuration in achieving higher performance targets.

For reference, the costs of the two-stage membrane process were outlined in a previous report and range from approximately 23 to 35 USD/t-CO_2_ [19]. A straightforward comparison is not feasible because of differences in these processes’ evaluation boundaries and parameters, such as the electricity prices used. However, it has been confirmed that the results are of comparable magnitude. Regarding the cost breakdown, it was previously indicated that changes in electricity prices had a greater impact on total costs than variations in membrane prices during a sensitivity analysis [18]. This analysis suggested that the electricity-related OPEX significantly contributes to total costs. It was also reported that even when the membrane price was set to the high level of 500 USD/m^2^, OPEX accounted for more than half of the total expenses, indicating that the membrane cost was not the primary contributor to the overall cost [19]. These previous reports suggest that the OPEX from electricity dominates these costs, which aligns with the findings of this study. Subsequent sections present a detailed analysis of the Pareto solutions and the interrelationships among design variables for each case study.

### 3.3. Membrane Area and Its Relationship to α*(CO_2_/N_2_), P_h_, and P_l_

**Figure 5:**
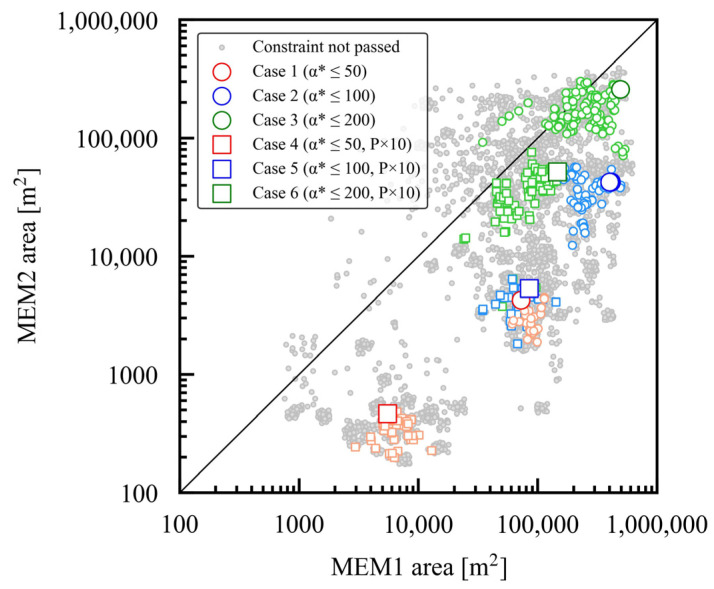
Optimized membrane areas for case studies 1 through 6, accounting for various membrane performance constraints. For each case study, the larger symbols represent the Pareto solutions, while the smaller symbols indicate the solutions that meet the constraints.

Figure 5 shows each case study’s optimal membrane areas for MEM1 and MEM2. MEM1 typically requires a larger membrane area than MEM2 due to its greater processing capacity. Earlier investigations into the two-stage membrane process with recycling concluded that the optimal solution required a first-stage membrane area about ten times larger than the second stage—a finding supported by the present study [16,18,19]. Case studies with higher maximum α*(CO_2_/N_2_) settings required larger membrane areas, highlighting the inherent trade-off between selectivity and permeability outlined by the Robeson upper bound. When comparing the case studies with the same maximum set value of α*(CO_2_/N_2_), the case studies with P × 10 require a membrane area that is smaller by approximately an order of magnitude, which corresponds to a tenfold increase in permeability.

**Figure 6:**
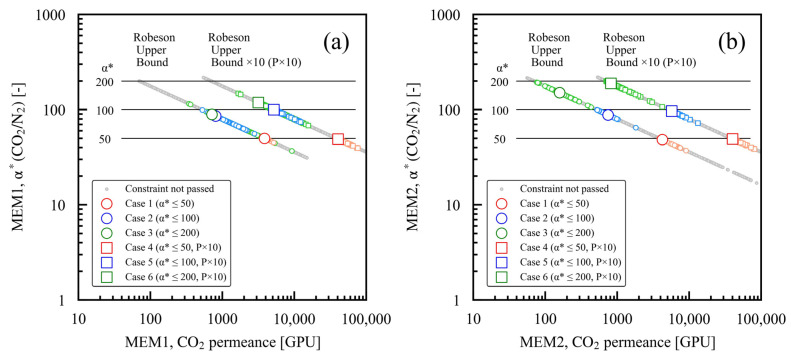
Optimized α*(CO_2_/N_2_) and CO_2_ permeance for case studies 1 through 6, considering various membrane performance constraints: (**a**) MEM1, (**b**) MEM2. In each case study, the larger symbols represent the Pareto solutions, while the smaller symbols indicate the solutions that meet the constraints.

The optimal performance of the membranes MEM1 and MEM2 in each case study is shown in Figure 6a,b, respectively. In cases 1 and 4, where the maximum setting value of α*(CO_2_/N_2_) was 50, the optimal value of α*(CO_2_/N_2_) for both MEM1 and MEM2 remained at 50. In case 2, where the maximum setting value of α*(CO_2_/N_2_) was 100, the optimal values of α*(CO_2_/N_2_) were approximately 86 for MEM1 and around 88 for MEM2. In case 5, the maximum setting value of α*(CO_2_/N_2_) was 100, and the optimal value of α*(CO_2_/N_2_) was approximately 100 for MEM1 and MEM2. In case 3, where the maximum setting value of α*(CO_2_/N_2_) was 200, the optimal values of α*(CO_2_/N_2_) were approximately 90 for MEM1 and approximately 150 for MEM2. In case 6, the maximum setting value of α*(CO_2_/N_2_) was 200. The optimal values were approximately 119 for MEM1 and about 190 for MEM2, both lower than 200 but higher than those in case 3. In cases 2 and 3, which featured Robeson upper bound setting, permeability decreases as α*(CO_2_/N_2_) increases, and a larger membrane area is required to achieve the same CO_2_ capture rate. As this situation is cost-prohibitive, a membrane value lower than the maximum set α*(CO_2_/N_2_) was selected. With the P × 10 setting added, the cost associated with the membrane area was low due to the membrane’s high permeability, and the maximum α*(CO_2_/N_2_) was chosen; even in case 5, where the maximum α*(CO_2_/N_2_) setting remained at 100. In case 6, where the maximum setting of α*(CO_2_/N_2_) was 200, a higher α*(CO_2_/N_2_) was chosen compared to that in case 3. Unlike the other case studies, in cases 3 and 6, the optimal value of α* was smaller for MEM1 and larger for MEM2. Consequently, the ratio of the area of MEM2 to MEM1 must exceed that of the other case studies, bringing the optimal solution closer to the diagonal in Figure 5.

**Figure 7:**
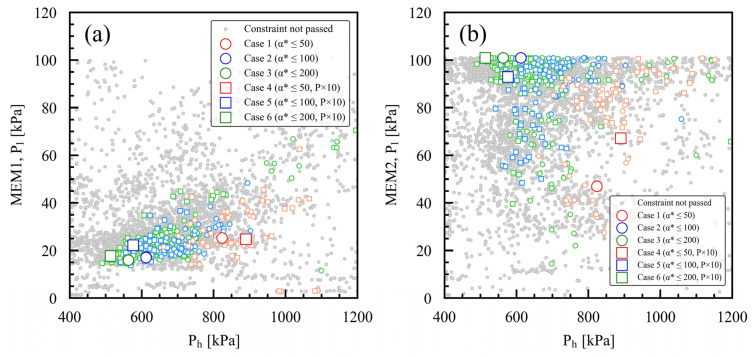
Optimized feed-side pressure (P_h_) and permeate-side pressure (P_l_) for case studies 1 through 6, considering various membrane performance constraints: (**a**) MEM1, (**b**) MEM2. In each case study, the larger symbols represent the Pareto solutions, while the smaller symbols indicate the solutions that meet the constraints.

Figure 7a,b illustrate the feed-side pressure (P_h_) and permeate-side pressure (P_l_) of MEM1 and MEM2 for each case study, respectively. In this study, the feed-side pressures of MEM1 and MEM2 are the same. In the case studies where the maximum setting values of α*(CO_2_/N_2_) were 50, 100, and 200, the feed-side pressures were approximately 800–900 kPa, 600 kPa, and 500–600 kPa, respectively. In case studies with the same maximum α*(CO_2_/N_2_) setting, the feed-side pressure was lower when P × 10 was considered. This is because permeability is high under P × 10 settings, allowing the required permeation to be achieved even with a low supply pressure. For MEM1, as illustrated in Figure 7a, the optimal permeate-side pressure was around 20 kPa for all case studies. For MEM2, as illustrated in Figure 7b, the optimal permeate-side pressure was 47 kPa in case 1 and 67 kPa in case 4, with these higher values indicating its greater permeability. The optimal permeation-side pressure was nearly the same for cases 2, 3, 5, and 6, at about 100 kPa. As described above, the bi-objective optimization algorithm was employed to investigate the optimal membrane area and pressure conditions for membrane performance, while considering the Robeson upper bound trade-off, based on the membrane performance settings of each case study.

### 3.4. Relationships Between Dimensionless Numbers α*(CO_2_/N_2_) and Pr and the Stage Cut

**Figure 8:**
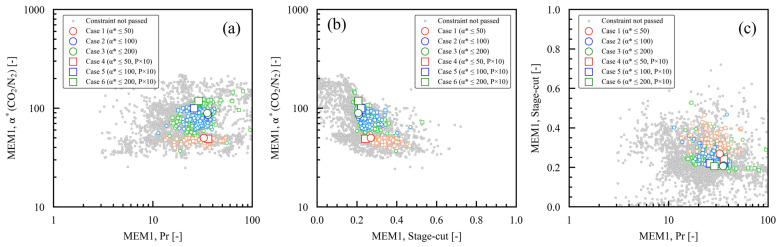
Dimensionless numbers for MEM1 case studies 1 through 6, which have varying membrane performance constraints: (**a**) α*(CO_2_/N_2_) and Pr, (**b**) α*(CO_2_/N_2_) and stage cut, (**c**) stage cut and Pr. In each case study, the larger symbols represent the Pareto solutions, while the smaller symbols indicate the solutions that meet the constraints.

**Figure 9:**
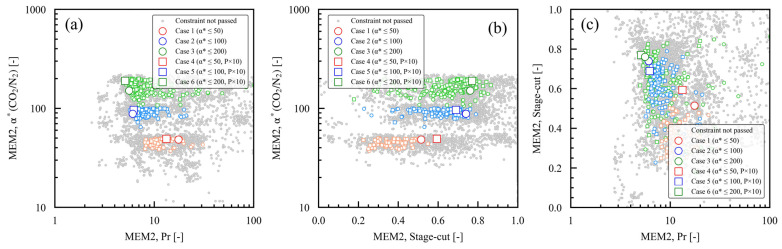
Dimensionless numbers for MEM2 case studies 1 through 6, which have varying membrane performance constraints: (**a**) α*(CO_2_/N_2_) and Pr, (**b**) α*(CO_2_/N_2_) and stage cut, (**c**) stage cut and Pr. In each case study, the larger symbols represent the Pareto solutions, while the smaller symbols indicate the solutions that meet the constraints.

Figure 8 and Figure 9 summarize the dimensionless numbers α*(CO_2_/N_2_) and Pr and the stage cut for each membrane module of MEM1 and MEM2, and their relationships. In Figure 8a, the Pr of MEM1 spans a range of approximately 20–40, demonstrating no clear trend throughout the case studies. In Figure 9a, the Pr of MEM2 is approximately 10–20 in cases 1 and 4, where α*(CO_2_/N_2_) is low, greater than the Pr 5–6 seen in the other case studies. Increasing the pressure ratio, Pr, is essential to achieving a high CO_2_ fraction on the permeate side when the membrane selectivity is low [15]. In cases 1 and 4, where the maximum α*(CO_2_/N_2_) is 50, the optimal conditions for meeting the constraint on the permeated CO_2_ fraction involve a high feed-side pressure (P_h_) and a low permeate-side pressure (P_l_), as shown in Figure 7b. This configuration ensures that the Pr is high in MEM2, which yields the desired product CO_2_. In Figure 8b, the stage cut of MEM1 is distributed within the range of approximately 0.2 to 0.3, showing no clear trend across the case studies. In Figure 9b, the stage cut of MEM2 is approximately 0.5–0.6 in cases 1 and 4, where the α*(CO_2_/N_2_) setting is low; about 0.7 in cases 2 and 5; and roughly 0.8 in cases 3 and 6. The higher the α*(CO_2_/N_2_) setting, the greater the stage cut. When membrane selectivity is low, the stage cut must remain small to satisfy the constraints on the permeated CO_2_ fraction [47]. For the case studies with a low α*(CO_2_/N_2_) setting, the optimum value was the smallest value for the stage cut of MEM2 that allowed the product CO_2_ to be obtained. In Figure 8c, the optimal values of the stage cut and Pr for MEM1 can be found in nearly the exact same location. In Figure 9c, the optimal values of the stage cut and Pr for MEM2 are higher, while the stage cut values are lower in case studies with lower maximum setting values for α*(CO_2_/N_2_). As mentioned previously, the relationship between the dimensionless numbers α*(CO_2_/N_2_) and Pr and the stage cut indicates that the design variables were optimized to lower costs and CO_2_ emissions while adhering to the constraints of the optimization process.

### 3.5. Relationships Between Membrane Performances and Membrane Area and Power Consumption

**Figure 10:**
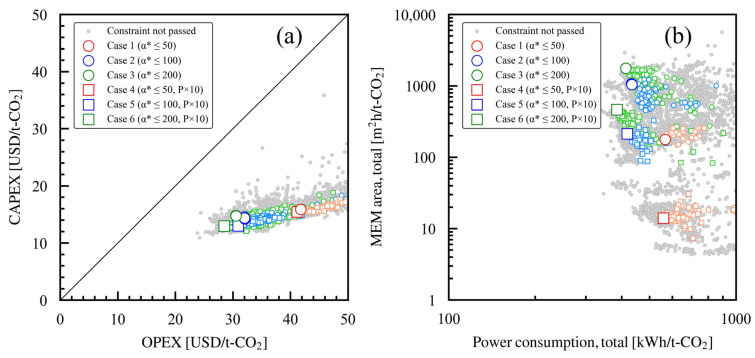
Comparison of the elements in the Pareto solutions: (**a**) CAPEX and OPEX across case studies 1 through 6, which have varying membrane performance constraints. (**b**) Membrane area and power consumption seen in Pareto solutions for case studies 1 through 6, which have varying membrane performance constraints. In each case study, the larger symbols represent the Pareto solutions, while the smaller symbols indicate the solutions that meet the constraints.

Further analyses were performed by categorizing the costs into OPEX (the costs derived from utilities) and CAPEX (the costs derived from equipment). In Figure 10a, these costs are contrasted by plotting CAPEX against OPEX. The diagonal line indicates the point where CAPEX and OPEX are equal. Cases where CAPEX dominates are positioned above this line, while cases where OPEX dominates lie below it. The Pareto solution is positioned below the diagonal line, indicating a higher proportion of OPEX than CAPEX in the overall cost of each case study. When comparing the OPEX and CAPEX values for each case study, the OPEX values increased significantly in cases 1 and 4, where the maximum setting value of α*(CO_2_/N_2_) was low, resulting in a high proportion of OPEX; this is because, as previously mentioned, in case studies where the maximum setting value of α*(CO_2_/N_2_) is low, the Pr must be increased and the stage cut reduced to meet the constraints on the permeated CO_2_ fraction.

The total membrane area and the power consumption per unit of produced CO_2_ were analyzed and are illustrated in Figure 10b. Lower maximum setting values of α*(CO_2_/N_2_) are associated with smaller total membrane areas and greater power consumption. The smaller the maximum setting value of α*(CO_2_/N_2_), the more power is consumed. As explained above, this is due to a large Pr, a small stage cut, and the increasing power required to generate the pressure required for operation. The case studies with identical maximum α*(CO_2_/N_2_) parameters present reduced membrane area when P × 10 is introduced. In case 6, the power consumption and membrane area are slightly smaller when compared to case 3, as the optimal solution utilized membranes with higher α*(CO_2_/N_2_) values in both MEM1 and MEM2, which reduces the power required for operation at the appropriate pressure, as illustrated in Figure 6a,b.

To summarize the trends seen, when the maximum set value of α*(CO_2_/N_2_) is high and a membrane with low permeability is used, the power required for the system’s operation decreases. The increase in cost due to the additional membrane area remains negligible. The membrane area can be reduced using a membrane with high permeability rather than a low maximum α*(CO_2_/N_2_) setting. However, the Pr must be increased and the stage cut must be decreased, which leads to greater power requirements. This, in turn, results in a higher OPEX due to electricity costs, the pump’s CAPEX, and CO_2_ emissions from electricity. As described above, the design variables were adjusted in the bi-objective optimization to minimize costs related to OPEX, CAPEX, and CO_2_ emissions from utilities.

### 3.6. Optimal Membrane Performance and Directions for Membrane Development

**Figure 11:**
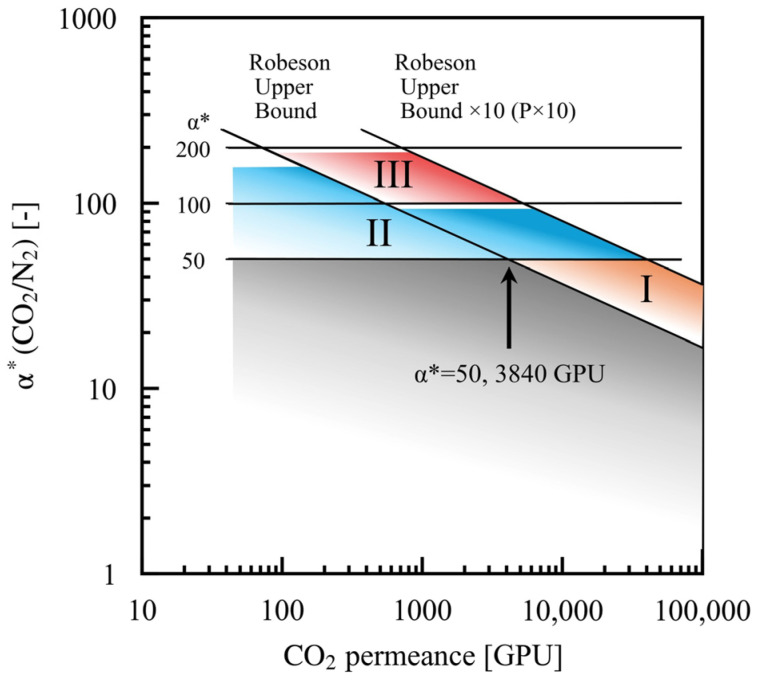
The direction in which the optimal membrane performance developed under the conditions assessed in this study. Membrane performance categories I, II, and III correspond to the enhancements in process performance shown in Figure 3.

This study explores the trajectory of the enhancements seen in membrane performance based on our case studies and the optimal Pareto solutions obtained. When comparing the optimal membrane performances across the trade-off between the Robeson upper bound (cases 1, 2, 3) and P × 10 (cases 4, 5, 6), as shown in Figure 6, a significant improvement in process performance is observed (as visible in Figure 3) as α*(CO_2_/N_2_) increases, despite a decrease in permeability from cases 1 and 4. This means that an improvement in α*(CO_2_/N_2_) has a greater impact on improving the process than an improvement in membrane permeability. The membrane performance categories are classified based on the case studies and displayed in Figure 11, and the corresponding maximum improvements in process performance are illustrated in Figure 3. The base case in this study corresponds to case 1, which has an α*(CO_2_/N_2_) set at 50 and a permeance of 3840 GPU. Membrane performance category I relates to case 2, where the membrane’s permeability improves from the baseline while α*(CO_2_/N_2_) is kept at 50. However, the improvement in the process performance is minimal, as shown in Figure 3. Membrane performance category II includes cases 3, 4, and 5. The objective of enhancing the membrane’s performance is to raise α*(CO_2_/N_2_) to 150, which is in alignment with the Robeson upper bound, or to improve α*(CO_2_/N_2_) to approximately 90 when P × 10 is considered, which is expected to result in a substantial improvement in process performance, as illustrated in Figure 3. Membrane performance category III includes case 6, where the membrane performance is enhanced to α*(CO_2_/N_2_) 190 with P × 10, indicating that further improvements in process performance are anticipated, as depicted in Figure 3. When constructing a two-stage membrane process using the developed membrane, the method for ensuring the optimal performance of the membrane in both the first and second stages is as presented in Figure 6. As described above, the comparison of the case studies evaluated in this research indicates that enhancing the membrane’s performance leads to a more significant improvement in process performance; increasing α*(CO_2_/N_2_) to categories II and III leads to a greater improvement than boosting permeability from the baseline case to category I.

Previous reports have indicated that improving selectivity has a greater impact when enhancing the performance of a membrane than increasing permeability [48]. The range of optimal membrane performances proposed by Merkel et al. encompasses categories I and II, as well as the baseline membrane performance, of the case studies considered in this study [14]. The optimal membrane performance range of the two-stage membrane-based separation suggested by Xu et al. covers categories I and II in this study during its first stage and had a range related to category III in its second stage [18]. As discussed above, this study outlines a direction for the improvement of membrane performances that aligns with previous proposals. Moreover, the analysis in this study provides precise numerical targets by considering the trade-off between permeation rate and selectivity.

## 4. Implications and Limitations

The membrane-based CO_2_ separation process is a promising method for capturing CO_2_; however, its optimization has not been fully explored due to the numerous design variables involved. Previously, optimization of this system specifically lacked a sufficient analysis of a bi-objective optimization related to cost, which is essential for implementing this process, and CO_2_ emissions, which are a vital part of CCUS. Additionally, for CO_2_ separation membranes whose performance is still being improved, there are insufficient quantitative targets for performance enhancements related to selectivity and permeability. Our research is the first to implement bi-objective optimization in a two-stage membrane-based CO_2_ separation process that includes recycling, taking into account both cost and CO_2_ emissions. The analysis has clarified the characteristics of the Pareto solutions and the influence of design variables on the final evaluation indices. It also confirmed that improving selectivity has a greater impact on membrane performance than enhancing permeability. The findings of this study are anticipated to improve the optimization of membrane-based CO_2_ separation processes and expedite the development of innovative membranes, thereby making membrane-based processes more competitive.

On the other hand, the process flow examined in this study was restricted to a two-stage membrane-based CO_2_ separation process with recycling and cannot be directly applied to other process flows. Additionally, since the concentration and flow rate of the supply gas are fixed, the optimal solution differs for various exhaust gas sources and processing flow rates, making our method not directly applicable in these cases. In the future, the methods used in this study are expected to be used to enhance the analysis of other membrane-based separation processes that involve various exhaust gas sources, and treatment scales, leading to advancements in the design of more practical and optimized membrane processes.

## 5. Conclusions

In this study, we carried out a bi-objective optimization of the costs and CO_2_ emissions of a two-stage membrane-based CO_2_ separation process that captures flue gas from a coal-fired power plant. Utilizing our original optimization algorithm, MLB-MOGABO, we created a dataset, developed ML models, searched for Pareto solution candidates, verified them, and repeated the process based on ADoE, conducting an optimization analysis with the constraints of a recovery rate of 0.9 and a purity of 0.95 for the CO_2_ produced. Regarding the membrane performance settings, six case studies were examined, in which the maximum setting value of α*(CO_2_/N_2_) was varied over three levels and the Robeson upper bound and a tenfold increase in permeability were also considered. The details of the Pareto solutions, the relationship between each design variable and the final evaluation index, and the impact of the membrane performance settings on the final evaluation index were clarified. The Pareto solutions for each case study indicated that as the maximum value of α*(CO_2_/N_2_) increases, both the cost and CO_2_ emissions decrease. More than half of the costs are OPEXs derived from electricity, which is directly reflected in the process’s CO_2_ emissions. This created a similar linear relationship between the costs and CO_2_ emissions, resulting in a single Pareto solution rather than a scenario where multiple solutions must be thoroughly examined across two axes. In each case study, the optimal performance of the membrane, the membrane area, the feed-side pressure, and the permeate-side pressure were improved based on the membrane’s performance settings, and the trends were summarized using dimensionless numbers. The greater the maximum value of α*(CO_2_/N_2_), the larger the optimal membrane area, which reflects the trade-off between selectivity and permeability made in the Robeson upper bound. In case studies with identical maximum α*(CO_2_/N_2_) settings, the P × 10 setting—which reflects the potential higher permeability of the membrane—required a smaller membrane area and lower feed pressure. Through a comparison of the case studies, it was clarified that advancements in membrane performance lead to a greater enhancement in process performance, by improving the α*(CO_2_/N_2_), than increasing permeability did. In future research, our analyses will include various other settings as external factors, such as different process flows, exhaust gas sources, treatment scales, and electricity prices.

## Figures and Tables

**Table 1:** Codes and types of equipment used, settings used for process simulation, and CAPEX evaluation settings applied to the process flow diagram shown in Figure 1.

Equipment Code	Equipment Type		Setting for Process Simulation	Setting for CAPEX Evaluation			
				Method	Capacity		
					Unit	Min.	Max.
C1, C2, C3	Compressor		Adiabatic efficiency: 75%.	CAPCOST [28], centrifugal, axial, and reciprocating	Fluid power, kW	450	3000
EX	Expander		Adiabatic efficiency: 75%.	CAPCOST [28], radial gas–liquid expanders	Fluid power, kW	100	1500
E1, E3	Heat exchanger		Hot side: gas; cold side: gas; solved with minimum internal temperature approach (ΔT: 10 °C); U-value determined by pressure [12].	CAPCOST [28], floating head	Area, m^2^	10	1000
E2, E4, E5	Heat exchanger		Hot side: gas (product temperature: 40 °C); cold side: cooling water (inlet temperature: 30 °C; outlet temperature: 40 °C); U-value determined by pressure [12].	CAPCOST [28], floating head	Area, m^2^	10	1000
MEM1, MEM2	Membrane separator	Membrane framework	Calculated based on the cross-flow model; pressure on both feed and permeate sides, membrane area, and permeance for all gas components involved in calculation.	Estimated based on previously published equation [22]; reference cost converted from EUR to USD at USD/EUR = 0.75	Area, m^2^	0	25,000
		Membrane module		Estimated by multiplying capacity and membrane module price	Area, m^2^	0	-

**Table 2:** Parameters used in the assessment of objective variables.

Parameter	Value	Unit	Remark
**CAPEX**			
Annual operation hour	8000	h/year	The annual production rate was calculated by multiplying the hourly production rate obtained from the process simulation by the annual operation hours
CEPCI_2001_	397.0	-	CEPCI for the year 2001 [28] was used as the base year
CEPCI_2014_	576.1	-	CEPCI for 2014 [31] was used as the base year for the evaluation of the membrane framework
CEPCI_2021_	708.0	-	CEPCI for year 2021 [31]
CRF_st_	0.098	-	Capital recovery factor calculated from service life: 25 years and interest rate: 0.08 used as standard values
CRF_MemModule_	0.250	-	Capital recovery factor calculated from service life: 5 years and interest rate: 0.08 used for membrane module
Membrane module price	50	USD/m^2^	-
**OPEX**			
Electricity	0.0718	USD/kWh	Average price in U.S. industrial sector in year 2021 [32]
Water	0.177	USD/1000 kg	[28]
**CO_2_ emissions factor**			
Electricity	0.656	kg-CO_2_/kWh	Determined by dividing the total CO_2_ emissions by the total amount of electricity generated from coal, natural gas, and petroleum, in the U.S. in 2021 [33]
Water	-	kg-CO_2_/1000 kg	Assessed using SimaPro for completely softened water [34]; value concealed in accordance with SimaPro’s terms and conditions

**Table 3:** Design variables and parameters used for bi-objective optimization.

Code	Design Variable	Unit	Setting for Bi-Objective Optimization			
			Range for Generating Sample Dataset in Iteration 1		Limit of Optimization Range	
			Min.	Max.	Min.	Max.
X1	MEM1 permeate side pressure	kPa	1	101	1	101
X2	MEM1 area	m^2^	1000	200,000	1	10,000,000
X3	MEM1 ideal separation factor, α*(CO_2_/N_2_)	-	10	200	10	1000
X4	MEM2 permeate side pressure	kPa	1	101	1	101
X5	MEM2 area	m^2^	1000	200,000	1	10,000,000
X6	MEM2 ideal separation factor, α*(CO_2_/N_2_)	-	10	200	10	1000
X7	C1 outlet pressure	kPa	200	2000	101	2000
X8	E1 area	m^2^	100	1000	1	100,000
X9	E3 area	m^2^	100	1000	1	100,000

**Table 4:** Objective variables, ML model building configurations, and bi-objective optimization parameters.

Code	Objective Variable	Unit	Remark	Set Used for ML Model Building		Set Used for Bi-Objective Optimization	
				Dataset	Method	Objective	Constraint
Y0	Convergence	-	Convergence of process simulation (1/0)	All data were used	RFC	-	=1
Y1	Product CO_2_ purity	-	Molar concentration of CO_2_ in the product	Only the converged data were used	GPR	-	≥0.95
Y2	Product CO_2_ recovery	-	Recovery of CO_2_ in the product			-	≥0.9
Y3	Cost	USD/t-CO_2_	Cost per t-CO_2_ in product			Minimize	-
Y4	CO_2_ emissions	t-CO_2_/t-CO_2_	CO_2_ emissions per t-CO_2_ in product			Minimize	-

**Table 5:** Configurations and outcomes of case studies.

Case Study Number	Setting		Result
	Ideal Separation Factor, α*(CO_2_/N_2_)	Membrane Permeability	Number of Pareto Solutions
1	≤50	Robeson Upper Bound	1
2	≤100	Robeson Upper Bound	3
3	≤200	Robeson Upper Bound	4
4	≤50	Robeson Upper Bound × 10 (P × 10)	1
5	≤100	Robeson Upper Bound × 10 (P × 10)	1
6	≤200	Robeson Upper Bound × 10 (P × 10)	1

## Data Availability

The original contributions presented in this study are included in the article. Further inquiries can be directed to the corresponding authors.

## Abbreviations

The following abbreviations are used in this manuscript:

**Nomenclature**	
CAPEX	capital expenditure, USD/t-CO_2_
CEPCI	chemical engineering plant cost index
CRF	capital recovery factor
C_TM_	total module cost, USD
F_BM_	bare module factor
OPEX	operation expenditure, USD/t-CO_2_
P_h_	feed-side pressure (absolute pressure), kPa
P_l_	permeate-side pressure (absolute pressure), kPa
Pr	pressure ratio (P_h_/P_l_)
**Equipment**	
C	compressor
E	heat exchanger
Ex	expander
MEM	membrane module
**Acronyms**	
ADoE	adoptive design of experiment
CAPCOST	capital equipment-costing program
CCUS	carbon dioxide capture, utilization, storage
GPR	Gaussian process regression
LCA	life cycle assessment
ML	machine learning
MLB-MOGABO	machine learning-based multi-objective genetic algorithm Bayesian optimization
NSGA-II	elitist nondominated sorting genetic algorithm-II
RFC	random forest classification
